# Ciltacabtagene autoleucel: The second anti-BCMA CAR T-cell therapeutic armamentarium of relapsed or refractory multiple myeloma

**DOI:** 10.3389/fimmu.2022.991092

**Published:** 2022-09-02

**Authors:** Endeshaw Chekol Abebe, Mestet Yibeltal Shiferaw, Fitalew Tadele Admasu, Tadesse Asmamaw Dejenie

**Affiliations:** ^1^ Department of Biochemistry, College of Health Sciences, Debre Tabor University, Debre Tabor, Ethiopia; ^2^ Department of Medicine, College of Health Science, Debre Tabor University, Debre Tabor, Ethiopia; ^3^ Department of Biochemistry, School of Medicine, College of Medicine and Health Sciences, University of Gondar, Gondar, Ethiopia

**Keywords:** CAR T-cell therapy, cilta-cel, multiple myeloma, efficacy, adverse effects

## Abstract

Ciltacabtagene autoleucel (also known as cilta-cel) is a chimeric antigen receptor (CAR) T-cell therapy that targets B-cell maturation antigen (BCMA) on the surface of cancer cells in B cell malignancies, such as multiple myeloma (MM). It is a second-generation CAR that is outfitted with an ectodomain comprising two BCMA-binding single chain variable fragment (ScFv) domains, a transmembrane domain, and an endodomain possessing CD3ζ and 4-1BB. Cilta-cel is an autologous, gene-edited CAR T-cell that is prepared by collecting and modifying the recipient’s T-cells to create a patient personalized treatment in the laboratory to be infused back. This CAR T-cell product exceptionally entails CARs with two BCMA-targeting single-domain antibodies that detect two epitopes of BCMA expressed on the malignant cells of MM. Cilta-cel is the current addition to the treatment armamentarium of relapsed or refractory (r/r) MM after its approval by the FDA on February 28, 2022, based on the results of the Phase 1b/2 CARTITUDE-1 study. It was the second approved anti-BCMA CAR T-cell product after idecabtagene vicleucel (ide-cel) to treat myeloma patients. It induces early, deep, and long-lasting responses with a tolerable safety profile in r/r MM. Cilta-cel-treated myeloma patients may potentially experience adverse effects ranging from mild to life-threatening, but they are mostly manageable toxicities. Besides, it has a consistent safety profile upon a longer follow-up of patients. Cilta-cel generally outperforms ide cel in terms of efficacy in MM, but shows comparable adverse events. This review highlights the current updates on cilta-cel efficacy, adverse events, comparison with ide-cel, and its future direction in the treatment of MM.

## Introduction

Chimeric antigen receptor (CAR) T-cell therapy is a novel advanced immunotherapeutic approach that targets specific tumor-associated antigens to kill cancer cells, slow disease progression, and increase patients’ survival and quality of life (1, 2). This therapeutic strategy is prepared by collecting the recipient’s T-cells and engineering them to create a patient-specific treatment that is then infused back into the patient to detect and kill antigen-expressing cancer cells (3). This technology is based on the idea that the body’s immune systems, particularly T-cells, are capable of recognizing and destroying cancer cells (4). The history of CAR T-cell therapy dates back three decades ago, and the field has evolved markedly from the first-generation CAR to the most recent fifth-generation CAR (5, 6). The CAR T-cell therapy has shown great advancements in terms of T-cell activation, proliferation, persistence, safety, and efficacy (7).

Many clinical trials around CAR T-cell therapy have been undertaken and are still being underway to develop effective therapeutic options for advanced cancers, with some demonstrating outstanding results (8). After arduous efforts, the Food And Drug Administration (FDA) has recently approved a few CAR T-cell therapies that have revolutionized the therapeutic outlook of a wide range of blood malignancies that are resistant to conventional therapies. Four CAR T-cell therapies targeting CD19 and two CAR T-cell treatments targeting B-cell maturation antigen (BCMA) have been approved so far by the FDA for the treatment of B-cell malignancies (9–11). Ciltacabtagene autoleucel is the most recently licensed anti-BCMA CAR T-cell product for the treatment of patients with advanced multiple myeloma (MM) (12). Herein, we aimed to rigorously review the status quo of the efficacy, potential toxicity, ongoing clinical trials, and future perspectives of ciltacabtagene autoleucel in MM treatment.

## Overview of BCMA-targeted CAR T-cell therapy in multiple myeloma

BCMA-directed CAR T-cell therapy is a novel treatment approach of MM that is manufactured by designing CARs of T-cells to detect and direct against the BCMA as an antigen. BCMA (CD269), also known as tumor necrosis factor superfamily member 17 receptor (TNFRSF 17), is a cell surface receptor that is exclusively presented on the surface of B-cell lineage cells (13, 14). TNFRSF17/BCMA interacts with member 13b of the TNF ligand superfamily, such as B-cell activating factor (BAFF) *via* the N-terminus BCMA TALL-1 binding domain and enhances plasma cell proliferation in the bone marrow (15, 16). Besides, BCMA binds with members of the tumor necrosis factor receptor-associated factor (TRAF) family and transduces signals for B-cell development, survival, proliferation, and differentiation into plasma cells. It is also deemed to be involved in the activation of nuclear factor-κB (NF-κB) and MAPK8/JNK (17).

Although BCMA is mainly expressed on the surface of B-cell lineage cells, including plasmablasts, differentiated plasma cells, and malignant plasma cells, its expression level is generally variable. While memory B-cells, naive B-cells, CD34 + hematopoietic stem cells, and other normal tissue cells do not present BCMA, cancerous B-cells express it much more than the healthy cells do (18, 19). The accumulated body of evidence showed that BCMA plays an important pathological role in the development of several hematological malignancies, such as MM (20, 21). BCMA is overexpressed in all MM and it is well established that it plays a key role in the pathogenesis of MM (21–23). Thus, BCMA is now considered the most popular and well-studied therapeutic target of CAR T-cell therapy in MM (21). Anti-BCMA CAR T-cell therapy is developed based on the concept that BCMA is preferentially expressed by plasma cells compared to other normal late-stage B cells, making it an ideal anti-tumor target in MM treatment (24).

In the last years, the FDA has approved two BCMA-targeting CAR T-cell products, namely idecabtagene vicleucel and ciltacabtagene autoleucel, which are designed to eradicate BCMA-expressing cells of MM (25, 26). On 26 March 2021, the first BCMA-targeted CAR T-cell therapy known as idecabtagene vicleucel (also named as Abecma™; ide-cel; bb2121) from Bluebird Bio has been authorized by the FDA for treating patients with relapsed or refractory(r/r) MM (26). Ide-cel was the first breakthrough in CAR T-cell development for the treatment of triple-class exposed adult patients suffering from r/r MM who received at least four prior lines of therapy (LOTs) (27). CAR T-cell therapy scene also hit another milestone in MM treatment when ciltacabtagene autoleucel was approved by the FDA on February 28, 2022, as the second BCMA-directed CAR T-cell therapy available commercially.

## Ciltacabtagene autoleucel in multiple myeloma

Ciltacabtagene autoleucel (also known as cilta-cel; Carvykti; JNJ-68284528; or LCAR-B38M CAR T-cells) is a gene-edited autologous CAR T-cell agent that expresses single-domain antibodies directed against two distinct epitopes of a BCMA target antigen (28). It is the most recently authorized CAR T-cell therapy as an alternative treatment approach for adult patients with r/r MM. It is the sixth FDA-authorized CAR T-cell product (after tisa-cel, axi-cel, brexu-cel, liso-cel, and ide-cel) in the treatment of B cell malignancies. It is also the second approved BCMA targeting CAR T-cell (after ide-cel) in the treatment of MM. Cilta-cel was first announced and commercialized by the Pharmaceutical Companies of Janssen and Legend Biotech. A marketing authorization application has recently also been submitted to the European Medicines Agency (EMA) and is pending a decision for licensing of cilta-cel in the treatment of r/r MM. This section of the review discusses the structural construct, manufacturing process, mechanism of action, efficacy, and adverse effects of cilta-cel in the treatment of MM.

### Structural construct

**Figure 1:**
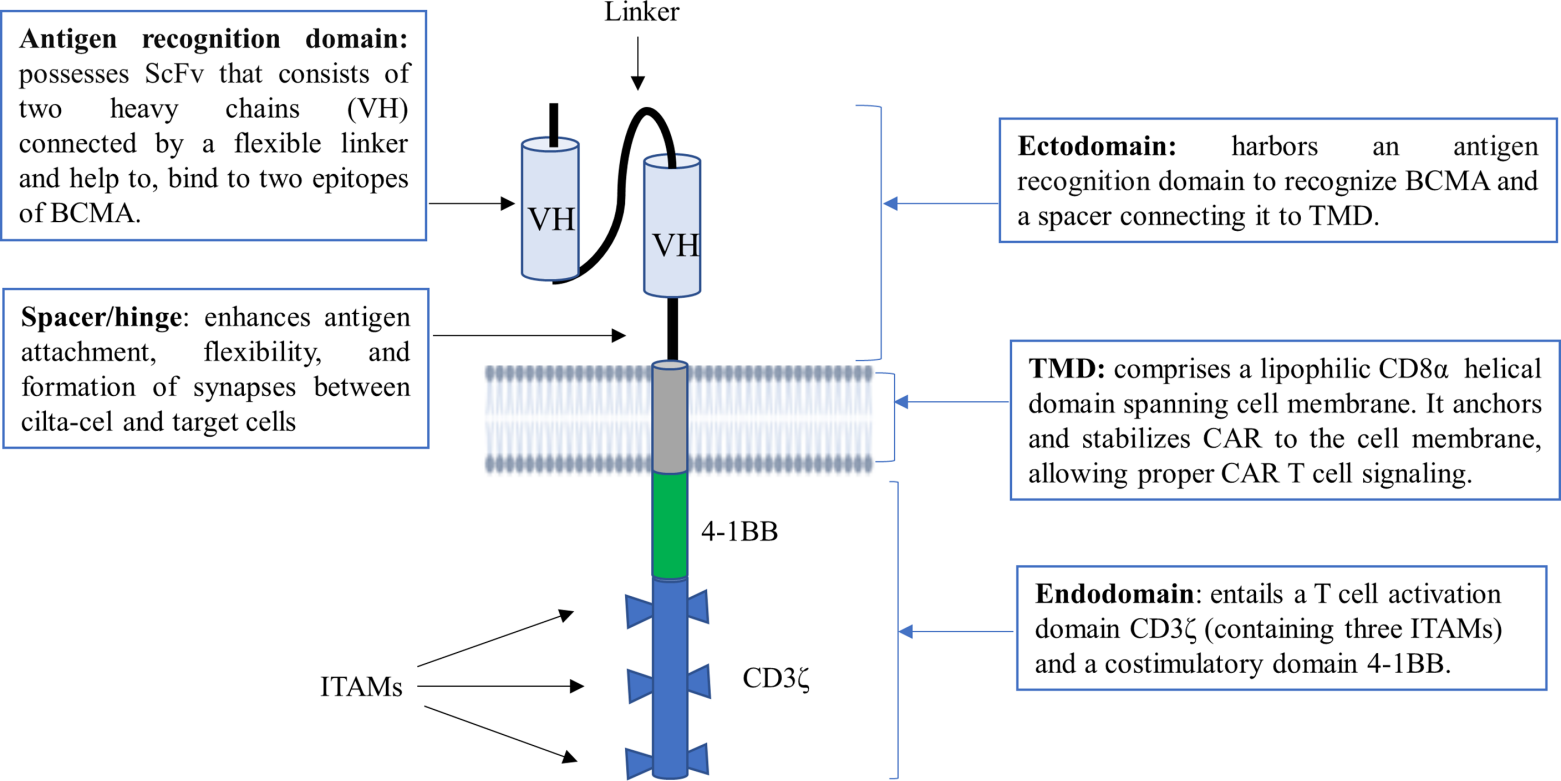
The structural construct of cilta-cel. It is a second-generation CAR T-cell containing an ectodomain, a TMD, and an endodomain. ScFv, short chain variable fragment; TMD, transmembrane domain; VH, variable heavy chain; BCMA, B cell maturation antigen; ITAM, immunoreceptor tyrosine-based activation motif.

Cilta-cel is a structurally differentiated second-generation CAR T-cell with synthetic receptors that bear the features of a monoclonal antibody (mAb) and a T-cell receptor (TCR) known as chimeric antigen receptor (CAR) (29). CAR protein comprises three principal components, namely an ectodomain, a transmembrane domain (TMD), and an endodomain (**Figure 1**) (30, 31). The ectodomain, also called the extracellular domain, consists of dual single-domain mAbs binding to two distinct BCMA epitopes. In other words, its antigen recognition domain typically possesses two llama (camelid) heavy chains (VH) as a single chain variable fragment (scFv) to bind with two epitopes of BCMA, providing high avidity against human BCMA (30, 32).

The ectodomain also possesses a spacer or hinge region that connects the scFv with the TMD. The spacer enhances antigen attachment, flexibility, and the formation of immunological synapses between CAR T-cells and target cells. The spacer can also be tuned to normalize the synapse distance between CAR T-cells and cancer cells (33). The TMD of CAR, which is commonly derived from CD8α, is found between the ectodomain and endodomain. It is important to anchor and stabilize CAR to the cell membrane, allowing proper CAR T-cell signaling (7, 31). On the other hand, an endodomain or a signaling cytoplasmic domain of cilta-cel is designed to entail a T-cell activation domain CD3ζ and a costimulatory domain 4-1BB. CD3ζ contains three immunoreceptor tyrosine-based activation motifs (ITAMs) as core units to mediate primary signals from scFv of the CAR receptor for T-cell activation. Upon the engagement of CAR, a costimulatory molecule (CM) 4-1BB (also known as CD137) mediates secondary or costimulatory signals for T-cell proliferation and persistence (7, 34).

### Manufacturing process

**Figure 2:**
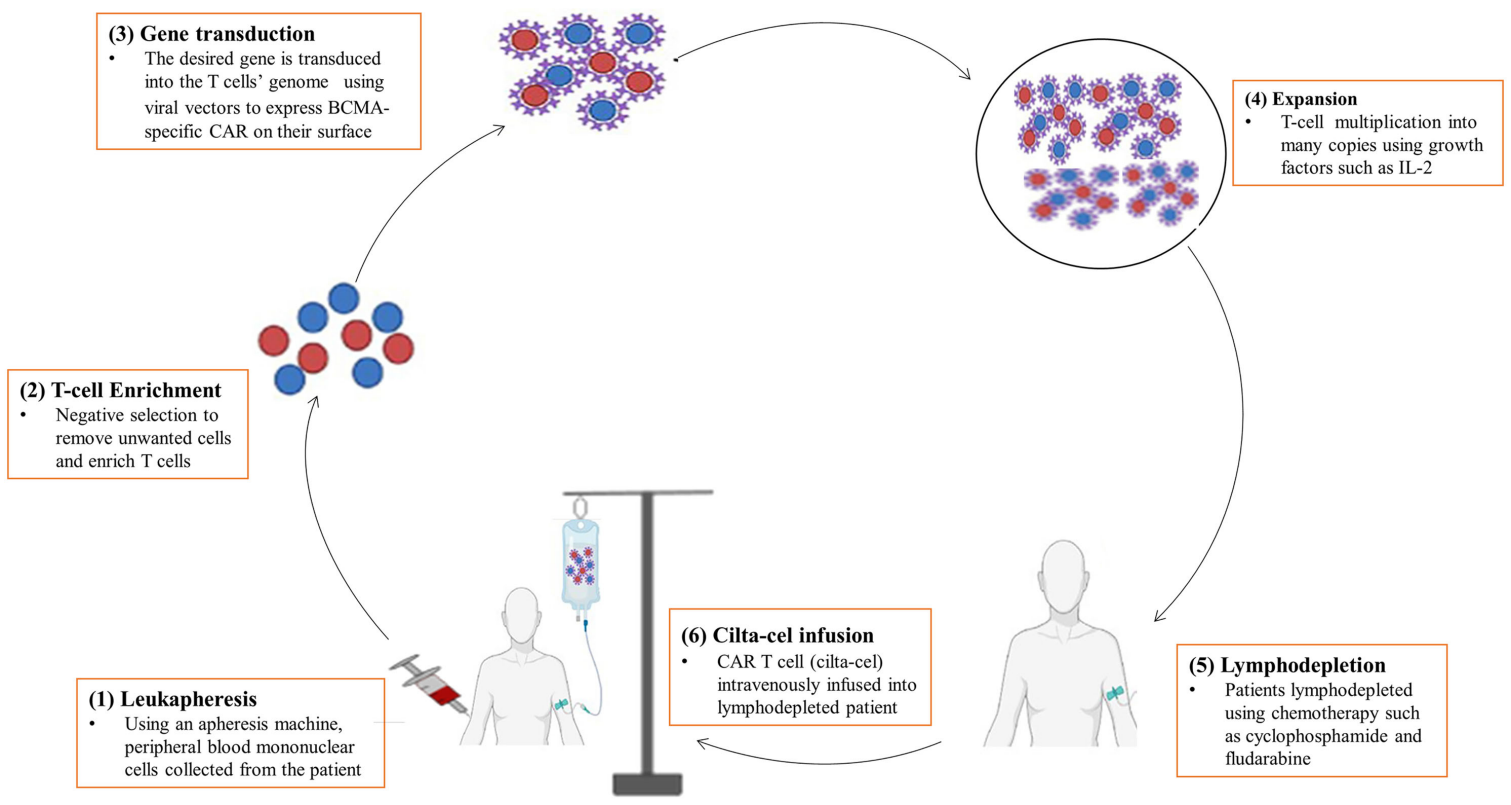
A diagrammatic representation of the manufacturing process of cilta-cel. It is prepared in the laboratory using several procedures, including (1) Leukapheresis, (2) T-cell enrichment, (3) Gene transduction, (4) Expansion, (5) Lymphodepletion, and (6) Cilta-cel infusion.

Cilta-cel is prepared in the form of cell suspension for intravenous infusion of patients with MM. It is typically manufactured in a laboratory setting by genetically modifying T-cells to express CARs outfitted with mAbs that recognize particular tumor-associated antigens (BCMA) on the surface of cancer cells. The production of cilta-cel is a sophisticated and laborious process that may take up to two to four weeks to complete and administer to the patient (35). It is an autologous CAR T-cell for which the manufacturing process starts off with a leukapheresis procedure to collect a patient’s own peripheral blood mononuclear cells (**Figure 2**). Mononuclear cells are enriched for T-cells by negative selection to remove unwanted cells with tetrameric antibody complexes recognizing non-T-cells and dextran-coated magnetic particles (36, 37). Then, T-cells are genetically altered with the desired gene using viral transduction, mostly by lentiviruses, to express CAR (35).

After genetic engineering, immunophenotyping analyses are carried out to ensure a successful endowment of T-cells with CARs and to determine the cytokine and cytolytic profiles of the CAR T-cells. Then, CAR T-cells are allowed to undergo ex vivo proliferation to multiply into millions of copies in the bioreactor vessel containing growth factor enrichment media. Growth factors and cytokines, such as IL-2, IL-7, IL-15, IL-21, and TGF-β1 can be used to promote the proliferation and differentiation of CAR T-cells (38–40). Finally, the CAR T-cell product (or cilta-cel) will then be isolated through purification followed by cryopreservation in the laboratory until it is given to the patient or immediately transported to the clinic for infusion. Intravenous reinfusion of cilta-cel into the recipient’s blood is done after lymphodepletion of patients with chemotherapy such as cyclophosphamide and fludarabine (35).

### Mechanism of action

**Figure 3:**
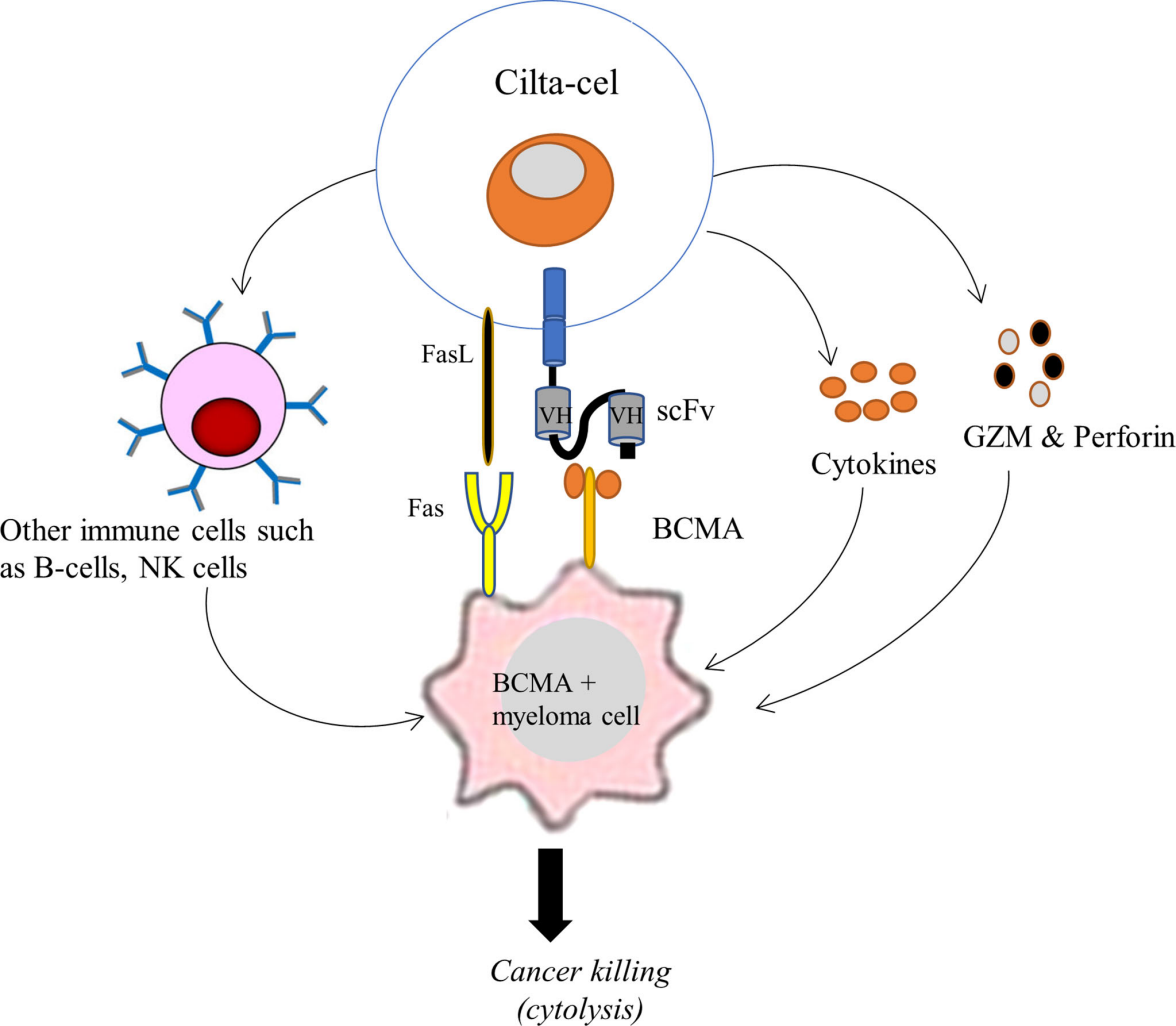
Schematic illustration of the mechanism of action of cilta-cel against BCMA expressing MM. Cilta-cel produces pro-inflammatory cytokines and recruits other immune cells (such as NK cells and B-cells) to the tumor site and induces inflammation that destroys myeloma cells. It also mediates the cytolysis of cancer cells by using an apoptosis-inducing perforin-granzyme system and Fas-FasL-axis. BCMA, B cell maturation antigen; FasL, Fas ligand; GZM, granzymes; NK cells, natural killer cells; VH, variable heavy chain.

Cilta-cel is designed to detect and eradicate BCMA-expressing cancer cells of MM. Following its intravenous infusion, cilta-cel seeks out malignant cells of MM and leads to an immune response triggering cytokine release (35). Through its CARs, cilta-cel first binds to BCMA positive cancer cells of MM and induces T-cell activation, expansion, and cancer cell elimination. The scFv (or mAb) of cilta-cel is responsible for human leukocyte antigen (HLA)-independent binding to a BCMA expressed on the surface of the cancer cell, leading to the activation of downward signaling proteins, including CD3ζ and CMs, within the CAR T-cell. CDζ transmits the primary signal, mimicking TCR signaling, for T-cell activation that induces extensive *in vivo* T-cell proliferation and differentiation. These CAR T-cells inside the patient’s blood play essential effector functions against cancer cells. They produce pro-inflammatory cytokines (such as TNF-α, IFN-γ, IL-2, and IL6) to induce inflammation that destroys cancer cells and recruit other immune cells (such as NK cells and B-cells) to the tumor site (**Figure 3**). Besides, cilta-cel mediates cytolysis of cancer cells by employing an apoptosis inducing perforin-granzyme system and Fas-FasL-axis (35, 41). This therapy is therefore important to reestablish the patient’s immune system and eradicate tumor cells. Moreover, 4-1BB (CD137) of cilta-cel transmits secondary signals to ensure cell survival and persistence for a longer period of time (35).

### Therapeutic efficacy

An accumulated body of clinical data reports that cilta-cel has remarkable therapeutic efficacy in patients with r/r MM, as indicated by different estimators of efficacy outcomes, including overall response rate (ORR), complete response or better (≥CR) rate, duration of response (DoR), progression-free survival (PFS), and overall survival (OS) (**Table 1**). Cilta-cel was initially evaluated by the first-in human clinical trial known as the phase 1 LEGEND-2 trial. The LEGEND-2 trial (NCT03090659) wa**s** a single-arm, open-label, multicenter phase 1 study in China that aimed to assess the safety and efficacy of cilta-cel among 57 patients with r/r MM as defined by the International Myeloma Working Group (IMWG) criteria and the National Comprehensive Cancer Network criteria (49, 50). After lymphodepleting chemotherapy was administered in the form of cyclophosphamide (300 mg/m2), three separate infusions of cilta cel (median dose:0.5 × 10^6^ CAR+ T-cells/kg; range, 0.07-2.1 × 10^6^ CAR+ T-cells/kg) were given. The results indicated that a single infusion of cilta-cel yielded early, deep, and sustained responses with an acceptable safety profile in heavily pretreated r/r MM patients. The data reported that 88% ORR, 68% CR, 5% very good partial response (VGPR), and 14% a partial response (PR), with 63% of patients achieving a negative rate of minimal residual disease (MRD) at 10^-5^. The median time to the first response was 1 month, while median PFS and DoR were 15 months and 14 months, respectively, at a median follow-up of 8 months. But the median OS was not reported (42).

**Table 1:** A summary table on the efficacy and adverse effects of cilta-cel in different clinical trials from their respective updated reports.

Clinical trials	Legend-2 trial (42)	CARTITUDE-1 (43)	CARTITUDE-2cohort-A (44–46)	CARTITUDE-2 cohort-B (47, 48)
Type of MM	r/r MM as defined by IMWG	r/r MM with ≥3 prior LOT	Lenalidomide refractory progressive disease after 1-3 prior LOT	Early relapse of MM after front-line therapy (a PI and IMiDs)
Patients treated	57	97	20	19
Median follow up duration (most recent)	8 months	18 months	14.3 months	13.4 months
Lymphodepleting agent	300 mg/m^2^ Cy	300 mg/m^2^ Cy and 30 mg/m^2^ Flu daily for 3 days	300 mg/m^2^ Cy and 30 mg/m^2^ Flu daily for 3 days	300 mg/m^2^ Cy and 30 mg/m^2^ Flu daily for 3-5 days
Target dose	Median: 0.5 × 10^6^ CAR+ T-cells/kg; range, 0.07-2.1 × 10^6^ CAR+ T-cells/kg	Median: 0.75×10 6 CAR+ T-cells/kg; range: 0.5-1.0×10^6^ CAR+ T-cells/kg	Median: 0.75×10^6^ CAR+ viable T-cells/kg	Median: 0.75×10^6^ CAR+ viable T-cells/kg; range: 0.5-1.0×10^6^ CAR+ viable T-cells/kg
ORR	88%	97.9%	95%	100%
≥CR rate	68%	80.4%	85%	90%
≥VGPR	5%	94.8%	90%	95%
MRD negativity at 10^-5*^	63%	91.8%	92.3%	93%
DoR	14 months	21.8 months	NR	NR
PFS	15 months	12 months in 66%	12 months in 84%	12 months in 90%
OS	NR	81%	NR	NR
Median time to first response	1 month	1 month	1 month	0.95 month
Median time to best response	NR	2.6month	3.3months	5.1 months
Median time to achieve ≥CR	NR	2.6 months	2.6 months	1.8 months
CRS of any grade	90%	94.8%	95%	84.2%
≥Grade 3 CRS**	7%	4.1%	10%	5.5%
ICANS of any grade	1.8%	20.6%	15%	5.5%
≥Grade 3 ICANS***	0.0%	10.3%	0.0%	0.0%
Number of deaths	6	10	4	0
Phase/status	Phase 1; continued as CARTITUDE-1	Phase 1b/2; approved but still underway	Phase 2; ongoing	phase 2; ongoing

*MRD evaluable samples; **based on the combined Lee et al. and American Society for Transplantation and Cellular Therapy (ASTCT) CRS severity grading criteria; ***based on the Common Terminology Criteria for Adverse Events (CTCAE) v5.0. ≥ CR, complete response rate or better; CRS, cytokine release syndrome; Cy, cyclophosphamide; DoR, duration of response; Flu, fludarabine; LOT, line of therapy; ICANS, immune effector cell-associated neurotoxicity syndrome; IMWG, International Myeloma Working Group; MRD, Minimal residual disease; NR, not reached/reported; OS, overall survival; ORR, overall response rate; PFS, progression-free survival; ≥VGPR, very good partial response or better; r/r MM, relapsed or refractory multiple myeloma.

The LEGEND-2 results also indicated a promising antimyeloma activity of cilta-cel in r/r MM patients with extramedullary disease (EMD) (42, 51). However, when compared to MM patients without EMD, patients with EMD generally demonstrate a poor prognosis and may lose their best response in a shorter period of time. The ORR in the EMD group was 82.4%, while the ORR in the non-EMD group was 90%. But there was no significant difference in the median time between the first response and the best response between the two groups. The median PFS in the EMD group and the non-EMD group was 8.1 months and 25 months, respectively. Whereas, the median OS in the EMD group was 13.9 months. Nonetheless, with 25 months of median follow-up, cilta-cel showed a relatively long-lasting therapeutic efficacy in r/r MM patients with EMD (51).

Following its exciting outcome in the treatment of r/r MM, the analysis of cilta-cel efficacy was continued in another geographical cohort in the US, which was jointly funded by Janssen and Legend Biotech, known as the CARTITUDE-1 phase 1b/2 trial (NCT03548207) (52). The Phase 1b/2 CARTITUDE-1 is an ongoing, single-arm, open-label, multicenter clinical study involving 16 centers in the US and enrolled 97 patients to evaluate the safety and efficacy of cilta-cel in adults with r/r MM who received ≥3 prior LOT, including a proteasome inhibitor (PI), an immunomodulatory drugs (IMiDs), and anti-CD38 mAb (12). The findings from CARTITUDE-1 trial are in line with those of the phase 1 LEGEND-2 study, proving that cilta-cel has resulted in excellent outcomes with tolerable safety profiles in r/r MM patients heavily pretreated with at least three standard LOTs (43).

The combined phase 1b and phase 2 of CARTITUDE-1analysis revealed that a single low-dose infusion of patients with cilta-cel (target dose 0.75×10 6 CAR+ viable T-cells/kg; range: 0.5-1.0×106 CAR positive viable T-cells/kg) following lymphodepletion using 300 mg/m^2^ cyclophosphamide and 30 mg/m^2^ fludarabine on daily basis for 3 days induces early, deep, and durable responses in patients with MM who have had more than three previous LOT (52, 53). Among 97 treated patients, cilta-cel elicited an ORR of 97.9%, a stringent complete response rate (sCR) of 80.4%,and >VGPR of 94.8%. The median time to first response was 1 month; the median time to best response was 2.6months; and the median time to >CR was 2.6 months, with 21.8 months of median DoR. Among 61 MRD evaluable patients, 91.8% were MRD negative at the 10^-5^ threshold, which was sustained for 6 months or above in 44.3% of patients (43). This indicates the greater cilta-cel efficacy (response rates) in the CARTITUDE-1 than in the LEGEND-2 trial, which could be due to the higher starting cilta-cel dose and the use of both cyclophosphamide and fludarabine as lymphodepleting agents in the CARTITUDE-1 than in LEGEND-1. Furthermore, a two-year follow-up data of CARTITUDE-1 showed long-lasting efficacy outcomes and over time deepening of cilta-cel’s therapeutic responses in the longer-term follow-up of MM patients. The findings indicated that cilta-cel continues to show a very high ORR (98%), with about 83% of those who received cilta-cel achieving above 67% sCR rate among responders. Further, 95% of patients achieved ≥VGPR with a 2-year PFS rate and OS rate of 61% and 74%, respectively (53). The outstanding efficacy and low adverse toxicity of cilta-cel observed in Legend-2 and CARTITUDE-1 studies propelled it further to receive FDA approval on 28 February 2022 as the second approved anti-BCMA-CAR T-cell product in treating triple class exposed MM (28).

Additionally, several clinical trials, including the CARTITUDE-2 trial, are now looking into the effectiveness of cilta-cel in the treatment of various MM population groups for which it is not yet licensed, as well as the feasibility of using it in an outpatient context. CARTITUDE-2 (MMY2003; NCT04133636) is an ongoing, open-label, multicohort (A-F), phase 2 clinical research examining the safety and efficacy of cilta-cel under various clinical settings for patients with MM, as well as evaluating the applicability of its use in an outpatient set up (44, 45, 47, 54). Only the data from cohorts A and B have been published so far, and both indicated promising results in different groups of MM patients. CARTITUDE-2 cohort A assessed 20 patients with lenalidomide refractory progressive MM who had received 1-3 prior LOTs, including a PI, an IMiD, and dexamethasone, and had never been exposed to any anti-BCMA agents. After 5–7 days of lymphodepletion of patients with 3 days of chemotherapy consisting of cyclophosphamide (300 mg/m^2^) and fludarabine (30 mg/m^2^), a single cilta-cel infusion with a target dose of 0.75×10^6^ CAR+ viable T-cells/kg was given to the patient (44–46). The results indicated that a single dose of cilta-cel infusion generally elicited early and deep responses in MM patients. The most updated results from this cohort have shown 95% ORR, 85% ≥CR, and 95% ≥VGPR after a median follow-up of 14.3 months among 20 cilta-cel-infused patients. Furthermore, the median time to first response was 1 month; the median time to best response was 3.3 months; and the median time to ≥CR was 2.6 months in this trial, although the median DoR was not reached. The 6-month and 12-month PFS rates were 90% and 84% respectively, with 92.3% of MRD-evaluable samples achieving MRD negative at 10^-5^ (45, 46).

The other cohort of CARTITUDE-2, cohort B, enrolled 19 MM patients who had undergone initial therapy with a PI and IMiDs and had disease progression for 12 months or less after frontline therapy but were not yet exposed to CAR T-cell therapy or anti-BCMA antibodies (47, 48). In CARTITUDE-2 cohort B, cilta-cel was administered at median target dose of 0.75×10 6 CAR+ viable T-cells/kg (range: 0.5-1.0×10^6^ CAR+ viable T-cells/kg) following lymphodepletion using 300 mg/m^2^ cyclophosphamide and 30 mg/m^2^ fludarabine on daily basis for 3-5 days. A 13.4-month median follow-up data from this trial indicated that a single target dose (0.75×10^6^ CAR+ viable T-cells/kg) of cilta-cel infusion achieved 100% ORR, 90% ≥CR, and 95% ≥VGPR in MM patients who were refractory to their prior LOT. Furthermore, the results showed that the first response was observed in a median time of 0.95 months, the best response was shown after 5.1 months of median time, and ≥CR was achieved at a median time of 1.8 months. Among fifteen MRD evaluable patients, 93% of them achieved to be MRD negative at 10^-5^. While the 12-month PFS rate was 90%, the median DoR was not reached (47).

### Adverse effects

Despite the successful outcomes and impressive remission rates of the cilta cel in the treatment of patients with r/r MM, accumulated evidence indicated that adverse effects that range from mild to life-threatening conditions may occur in treated patients (25). According to the phase-1 Legend-2 trial, adverse events were reported in all patients with r/r MM who received cilta-cel but they were largely manageable. The most common adverse events (incidence greater than 40%) of any grade were fever (91%), CRS (90%), thrombocytopenia (49%), and leukopenia (47%). Adverse effects with the severity of grade 3 or above were noted in 65% of patients. CRS was the most common grade ≥3 adverse event observed among treated patients, with about 7% of them were experienced grade ≥3 CRS (**Table 1**). The median onset and duration of CRS in cilta-cel-treated MM patients were 9 days. Only one patient (1.8%) developed neurotoxicity at 1.0x10^6^ CAR+ T-cells per kg cilta-cel dose, manifested with grade 1 neurological symptoms (such as aphasia, agitation, and seizure-like activity) that resolved within a day after treatment. A total of six patients died during follow-up due to disease progression and other causes (42).

In the previous years, multiple updates from the CARTITUDE-1 trial were made regarding cilta-cel safety in r/r MM. The cumulative data demonstrated that a consistent safety profile of cilta-cel and no new safety signals were observed with longer follow-up of myeloma patients. Based on the most updated CARTITUDE-1 report, the commonest adverse effects (incidence greater than 70%) of any grade observed during cilta cel use were CRS (94.8%), neutropenia (90.7%), anemia (81.4%), and thrombocytopenia (79.4%). Further, the report indicated that the most common grade ≥3 hematologic adverse (incidence rate >25% of patients) were neutropenia (94.8%), anemia (68.0%), leukopenia (60.8%), thrombocytopenia (59.8%), and lymphopenia (49.5%) (43). However, the main side effect associated with cilta-cel therapy was CRS, ranging from mild to life-threatening conditions that require careful monitoring and management. In CARTITUDE-1 reports, most of the patients who experienced CRS were either grade 1 or 2, with only 4.1% of CRS being grade≥3 (55). The median onset and median duration of CRS were 7 days and 4 days, respectively. CRS is generally treatable in most patients with r/r MM; 98.9% of them were resolved within 14 days of onset (43).

In addition, neurotoxicity events, which can be immune effector cell-associated neurotoxicity syndrome (ICANS) or non-ICANS, are associated with post cilta-cel infusion of MM patients. ICANS is a heterogenous condition that manifests with a highly variable clinical course, including aphasia, altered consciousness, cognitive skills impairment, motor weakness, seizures, and cerebral edema. The non-ICANS neurotoxicity events, which usually present with symptoms that do not fit the current definition for ICANS, involve a cluster of movement disorders (like micrographia, tremors, Parkinsonism), cognitive impairment (e.g., memory loss, disturbance in attention, amnesia, encephalopathy), and personality changes (such as. facial nerve palsy with reduced facial expression, flat affect). These cilta-cel-linked neurotoxic events warrant careful monitoring and timely management to avert potentially life-threatening or permanent neurologic sequelae (55, 56). According to the report of CARTITUDE-1, about 20.6% of patients developed neurotoxicity (both of any grade, with 10.3% of them being grade 3 or above. About 16% of patients experienced ICANS, mostly (14%) grade 1/2 but only 2% of patients were having a grade 3 and 4 event.

Other CAR T-cell neurotoxicities occurred in 12.4% of patients, including facial palsy, neurotoxicity, concentration impairment, diplopia, cranial nerve palsy, sensory loss, ataxia, peripheral motor neuropathy, and peripheral sensory neuropathy. Both ICANS and other non-ICANS neurotoxicities overlapped in 8.2% of patients treated with cilta-cel. The most updated data from CARTITUDE-1 indicated that about 1.5% of patients reported movement and neurocognitive treatment-emergent adverse events (TEAEs). TEAE is characterized by the presence of a combination of at least two of the following features: high tumor burden, grade ≥2 CRS or ICANS of any grade after cilta-cel infusion, and high CAR T-cell expansion/persistence. Mitigation strategies such as enhanced bridging therapy to reduce baseline tumor burden, early aggressive treatment of CRS and ICANS, handwriting assessments for early symptom detection, and extended monitoring/reporting time for neurotoxicity beyond 100 days post-infusion, have been implemented across the cilta-cel development program to prevent TEAEs in subsequent studies. Effective implementation of such strategies reduces the incidence of TEAEs from 5% to less than 1% across the cilta-cel program (57).

Further, CARTITUDE-1 study has reported ten post-cilta-cel infusion deaths, which are attributed to the disease’s progression in two patients and treatment-related adverse effects or other unrelated causes in eight MM patients (43). Despite its approval, cilta-cel use in the treatment of myeloma patients may lead to life-threatening adverse toxicities and thus requires precaution with an appropriate risk evaluation and mitigation strategies. Therefore, cilta-cel therapy requires hospitals with qualified and trained professionals to identify and manage adverse effects, such as CRS and ICANS. The FDA is also currently requesting the manufacturer to undertake a post-marketing observational study among cilta-cel-treated patients to assess long-term safety (28).

The CARTITUDE-2 clinical trial also reported the safety profile of cilta-cel in the treatment of other MM populations. According to the reports from the cohort-A of the CARTITUDE-2 study, the safety profile of cilta-cel in MM patient groups was manageable, involving hematologic adverse effects (≥20%): neutropenia (95%), thrombocytopenia (80%), anemia (75%), lymphopenia (65%), and leukopenia (55%). CRS occurred in 95% of treated patients, with a median onset of 7 days and a median duration of 4 days. But only 10% developed grade ≥3 CRS and it resolved within 7 days in 90% of patients. Neurotoxicity occurred in 20% of patients, but only 15% of patients experienced ICANS of all grades, with a median onset of 8 days and a median duration of 3-days. One patient had faced other neurotoxicity, grade 2 facial paralysis after 29 days of cilta-cel administration that lasted for 51 days. However, no movement and TEAEs were observed. Four post cilta-cel infusion deaths due to treatment-related or unrelated conditions were documented during the CARTITUDE-2 trial. One patient treated in the outpatient setting experienced similar safety outcomes as hospitalized patients, suggesting the possibility of using cilta-cel for outpatients (45).

Overall, cilta-cel in patients with progressive MM who received 1-3 LOT and were lenalidomide refractory showed a manageable safety profile and suitability to use in an outpatient setting.

On the other hand, cohort-B of the CARTITUDE-2 showed that 20% of patients or above have experienced treatment-associated hematological adverse events, involving neutropenia (88.9%), thrombocytopenia (61.1%), anemia (50.0%), leukopenia (27.8%), and lymphopenia (22.2%). The most recent updated report indicated that about 84.2% of all cilta-cel received patients developed CRS of any grade, with nearly 5.5% encountering grade 4. The median onset of CRS was 8 days, and its median duration was 4 days. About 5.5% of patients developed grade 1 ICANS and 5.5% of them experienced grade 3 TEAEs on day 38 of post cilta-cel infusion. But only one death has been reported on day 158 of post cilta-cel infusion (47, 48). The collective evidence from CARTITUDE-2 demonstrates an encouraging antimyeloma activity and a manageable safety profile of cilta-cel in different conditions of MM other than the approved ones. Thus, there is a great chance that cilta-cel will soon be licensed for medical use for those MM patient groups under the CARTITUDE-2 study, with the possibility of being used in outpatient settings.

## Comparison of ciltacabtagene autoleucel with idecabtagene vicleucel

Cilta-cel and ide-cel are anti-BCMA CAR T-cell therapies that are designed to identify and eradicate BCMA-expressing malignant plasma cells of MM. They both have similarities and differences in several aspects. This part of the review summarizes the comparisons between the two products based on their general features, efficacy, and adverse effects.

### General features

The FDA has approved cilta-cel and ide-cel for medical use as frontier LOTs for triple exposed r/r MM patients, based on the data from the KarMMa trial and phase 1b/2 CARTITUDE-1 study, respectively (26, 43). The ide-cel was first developed by Bluebird Bio (BB) and has been marketed by Bristol Myers Squibb (BMS), whereas the cilta-cel was first introduced and marketed by Janssen (25, 43). In terms of structure, they are genetically engineered autologous second-generation CAR T-cell agents with anti-BCMA antibodies in the ectodomain to direct against BCMA antigens, as well as a primary signal transmitter (CDζ) and a CM (4-1BB) in the endo-domain. However, ide-cel contains a single mouse-derived binding domain to target only one epitope of the BCMA antigen (25, 52). On the other hand, cilta-cel has a unique CAR design expressing two camelid heavy chains(VH) of mAbs to bind with two separate epitopes of BCMA antigen (29, 35). This makes cilta-cel a unique CAR T-cell agent that confers a higher avidity of binding to the target cells, enhanced activity, and lowered immunogenicity compared to ide-cel. As a result, the CAR T-cell dose may be reduced, which was anticipated to lower the occurrence of the adverse effects. It is unclear, nevertheless, whether this is related to the better depth and remission observed with cilta-cel. Furthermore, ide-cel is a one-time infusion, with a recommended dose ranging between 300-460×10^6^ viable CAR positive T-cells per kilogram of the body weight. Cilta-cel is also administered as a single infusion at a target dose ranging between 0.5-1.0 × 10^6^ CAR+ viable T-cells/kg (25). The comparison between the two agents in their general features is summarized in **Table 2**.

**Table 2:** Idecabtagene vicleucel versus ciltacabtagene autoleucel based on their general features.

Feature	Idecabtagene vicleucel	Ciltacabtagene autoleucel	Refer.
Brand name	Abecma™	Carvykti™	(28, 58)
Nick name	Ide-cel	Cilta-cel	(28, 58)
Design	Second generation	Second generation	(29, 35)
Ectodomain	One anti-BCMA	Two anti-BCMA	(29, 35)
Endo-domain	CDζ-4-1BB	CDζ-4-1BB	(29, 35)
Clinical study	KarMMa trial	CARTITUDE 1b/2	(12, 26)
Date of approval	26 March 2021	28 February 2022	(28, 58)
Developer company	Bluebird Bio and Bristol Myers Squibb	Janssen and Legend BioTech	(26, 43)
Therapy class	BCMA-directed CAR T cell	BCMA-directed CAR T cell	(28, 58, 59)
Indications	Triple class exposed r/r MM	Triple class exposed r/r MM	(26, 43)
Recommended dose	300-460×10^6^CAR+ T cells/kg	0.5-1.0 × 10^6^ CAR+ T cells/kg	(26, 43)

### Therapeutic efficacy

Based on their respective clinical trials, both cilta-cel and ide-cel show unprecedently high response rates and survival outcomes in r/r MM patients, which is higher efficacy than the conventional LOTs of MM (12, 25, 26, 43). An increasing body of evidence shows that both cilta-cel and ide-cel exhibit superior outcomes in triple-class exposed r/rMM when compared to three recently approved novel combination therapies (selinexor in combination with dexamethasone, belantamab mafodotin, and melphalan flufenamide). These novel combination therapies show a low ORR ranging from 26-34% and a limited PFS of less than 5 months (60–62). In contrast, multiple clinical studies showed that both ide-cel and cilta-cel significantly improved efficacy outcomes.

Ide-cel was approved in 2021 owing to its excellent efficacy and durable responses in the KarMMa trial (58). KarMMa trial (NCT03361748) was conducted to assess the safety and efficacy of ide-cel among 127 patients with r/r MM after four or more prior LOT (IMiD, a PI, and an anti-CD38 mAb). The results from this clinical study demonstrated that patients with advanced MM heavily treated using ide-cel had markedly improved responses, with approximately 73% ORR and a 33% CR rate (**Table 3**) (26). The data also showed improved survival outcomes of patients after ide-cel infusion, with an average of 8.6 months of PFS and 24.8 months of OS, and DOR of 10.9 months. MRD negativity at 10^-5^ was confirmed in 26% of patients. About 79% of patients who achieved a ≥CR or better were confirmed to show MDR negative status (25, 63). Whereas CARTITUDE-1 showed that cilta-cel induces deep and durable responses in triple-class exposed r/r MM patients, with 97.9% ORR, 80.4% sCR and 94.8% ≥VGPR. Besides, cilta-cel achieved a 12-month OS and PFS in 81% and 66% of patients, respectively, with a DoR of 21.8 months (43).

**Table 3:** Ide-cel versus cilta-cel based on their efficacy and adverse events in MM treatment based on KarMMa trial and CARTITUDE- 1.

CAR T cell	Study	Pts	ORR	≥CR	≥VGPR	MRD negativity at 10^-5*^	PFS	OS	DoR	Any grade CRS	Grade≥3 CRS	Any grade ICANS	Grade ≥3 ICANS
Ide-cel	KarMMa trial (25, 26, 63)	127	73%	33.1%	57.9%	26%	8.6mo.	24.8 mo.	10.9mo.	84%	5%	18%	3%
Cilta cel	CARTITUDE- 1 (43)	97	97.9%	80.4%	94.8%	91.8%	12mo. in 66%	12 mo. in 81%	21.8 mo.	94.8%	4.1%	20.6%	10.3%

*Among MDR evaluable patients; mo, months; pts, number of treated patients.

Based on the rough comparisons of the results of the KarMMa trial with those of CARTITUDE-1 phase 1b/2, ide-cel has lower efficacy in treating patients with r/rMM than cilta-cel. However, as the two studies were conducted in distinct cohorts, this naive comparison of the results from independent trials is unadjusted and unmatched, which may cause confounding bias and lead to an unequal comparison of their prognostic characteristics. The comparative efficacy of the two CAR T-cell therapies in treating triple exposed r/r MM was not assessed in the head-to-head clinical trial either. But a matching-adjusted indirect comparison (MAIC) of the efficacy outcomes for cilta-cel versus ide-cel in the treatment of triple class exposed r/r MM has recently been undertaken using individual patient-level data for CARTITUDE-1 and published summary-level results for KarMMa (64). The findings from the MAIC study confirmed that cilta-cel generally has superior efficacy in all outcomes (ORR, ≥CR, DoR, PFS, and OS) in r/r MM patients than ide-cel. Cilta-cel-treated patients had 1.3 times more likelihood to respond (ORR) and 2.2-times more odds of achieving CR or better when compared to ide-cel-treated MM patients after appropriate adjustment was made using the MAIC (64). Besides, cilta-cel is demonstrated to be associated with an increased likelihood of a deeper response, as indicated by the improved PFS and OS than ide-cel. The DoR of cilta-cel was also substantially higher than that of ide-cel (64–66).

### Adverse effects

Despite their impressive responses in MM patients, both cilta-cel and ide-cel are associated with adverse effects (67). The main symptoms in two CAR T-cell products involve CRS, ICANS, infections, fatigue, musculoskeletal pain, hematological adverse events, and hypogammaglobulinemia. In general, most CRS events in CARTITUDE-1 and KarMMa were not severe (25, 53). Based on the KarMMa study, CRS was reported in 84% of ide-cel-treated patients (**Table 3**). About 79% of cases of CRS were either grade 1 or grade 2, but only 5% of them were grade ≥3 (25). Similar results were reported in CARTITUDE-1, wherein 95% of patients experienced CRS, and the majority of CRS cases (95%) were grade 1 or grade 2 and 4.1% were grade ≥3 CRS (53). Furthermore, both products have been observed to differ in median onsets and durations of CRS. The median onset and duration of CRS in the ide-cel range between 1-2 days and 4–7 days, respectively. Whereas a median onset of 7 days and median duration of 4 days was reported in cilta-cel-infused patients (25, 53). In addition, KarMMa trials revealed that neurotoxic events (such as ICANS) have been shown in 18% of patients who received ide-cel and only 3% of them were grade ≥3 (25). On the other hand, 20.6% of cilta-cel treated patients developed ICANS with about 10.3% of them were grade ≥3. Neurocognitive and other non-ICANS related TEAEs were seen with cilta-cel while these have not been reported with ide-cel. Moreover, the most grade ≥3 hematologic toxic events in ide-cel treated myeloma patients, including neutropenia (89%), anemia (60%), and thrombocytopenia (52%). Whereas, the most common grade ≥3 hematologic adverse events in cilta-cel infused patients were neutropenia (94.8%), anemia (68.0%), leukopenia (60.8%), thrombocytopenia (59.8%), and lymphopenia (49.5%). But the incidence of all grade infections was similar between the two products (25, 43). Cilta-cel also showed a reduction in the risk of death by approximately 45% compared to ide-cel (64). Overall, although there was no head-to-head clinical trial or MAIC study that compares the safety profiles of cilta-cel and ide-cel in r/r MM patients, cumulative evidence showed that both have a comparable incidence and magnitude of treatment-associated adverse effects.

## Ongoing clinical trials on ciltacabtagene autoleucel

The field of CAR T-cell therapy in MM is quickly growing as a result of the promising results in earlier studies. There are currently more than 50 active clinical trials in various stages, CAR designs, and targets (68). Several target antigens of CAR T-cell therapy are undergoing intensive research in this disease, which may open a new arena in MM therapy. While certain antigen targets have had unfavorable outcomes due to side effects and poor efficacy, some other targets such as CD38, SLAMF7/CS1, or GPRC5D have shown encouraging results. Notably, BCMA is the most prominent and well-studied therapeutic target of myeloma. BCMA-directed CAR T-cell therapy, such as ide-cel and cilta-cel, has been demonstrated to be the most effective and safe in treating myeloma patients with high response rates and low rates of serious side effects (29, 35).

A prospective follow-up study phase 1b/2 CARTITUDE-1, which started a long time ago by recruiting 97 eligible patients and providing data that allowed cilta-cel approval, is still ongoing (**Table 4**). The updated longer-term follow-up data from this trial on the safety and efficacy of cilta cel in MM is anticipated in near future. Nevertheless, cilta-cel use with or without prior LOT in other diverse situations of MM, and its suitability for outpatient treatment have yet not been approved by regulatory agencies, such as the FDA. Thus, several clinical trials, in addition to CARTITUDE-1, are currently underway to further explore and update the efficacy outcomes and safety profiles of cilta-cel use alone or in combination with standard therapies in various conditions of MM other than those that have recently been received cilta-cel approval. Clinical trials such as CARTITUDE-2, CARTITUDE-4, CARTITUDE-5, and CARTITUDE-6 are currently ongoing to assess cilta-cel use in various MM types and outpatient settings.

**Table 4:** A summary table on the ongoing clinical trials assessing cilta-cel safety and efficacy in the treatment of MM under various conditions.

Clinical study	Trial number	Purpose	Clinical sites	Patient number	Phase/Status
CARTITUDE-1 (69)	NCT03548207	Evaluating the safety and efficacy of cilta-cel in adults with r/r MM who received ≥3 prior LOT (PI, an IMiDs, and anti-CD38 mAb)	16	97	Phase1b/2; data published; cilta-cel approved for this type of r/r MM, but still active
CARTITUDE-2 (70)	NCT04133636	Assessing cilta-cel efficacy and safety in patients who had progressive MM after 1-3 prior LOT and were refractory to lenalidomide (Cohort A), early relapse after initial therapy (cohort B), r/r MM after PI, anti-CD38 antibody, an IMiD, and BCMA-directed treatment (cohort C), Less than CR after ASCT front-line therapy (cohort D), NDMM with the high-risk disease after no or only one cycle of prior therapy (cohort E), NDMM with standard-risk (ISS stage I and II) and after initiation of therapy (cohort F)	45	157	Phase 2, data from cohort A and B are published (45, 47, 48), but they are still ongoing, while other cohorts are in progress (not yet recruited patients)
CARTITUDE-4 (71)	NCT04181827	Compare cilta-cel safety and efficacy versus standard LOTs in adult patients with relapsed and lenalidomide refractory MM.	100	400	Phase 3; Active but not yet recruiting
CARTITUDE-5 (72)	NCT04923893	Assess the efficacy and safety of cilta-cel as a frontline therapy in patients with NDMM not intended for transplant.	118	650	Phase 3; recruiting patients
CARTITUDE-6 (73)	NCT05257083	Compare the efficacy and safety of DVRd followed by a single infusion of cilta-cel versus DVRd followed by ASCT in patients with NDMM who are not exposed to prior BCMA targeted therapy.	52	750	Phase 3; Patients recruitment has not yet started

ASCT, autologous stem cell transplant; DVRd, daratumumab, bortezomib, lenalidomide, and dexamethasone; IMiDs, immunomodulatory drugs; ISS, international staging system; LOT, line of therapy; mAb, monoclonal antibody; MM, multiple myeloma; NDMM, newly diagnosed MM; PI, protease inhibitor.

CARTITUDE-2 trial (NCT04133636) is a phase 2 clinical trial that has begun in November 2019 by enrolling 157 participants in six cohorts (A to F). The study started with the aim of evaluating cilta-cel safety and efficacy for myeloma patients in a variety of clinical settings and to determine whether outpatient administration is feasible. Among all cohorts of CARTITUDE-2, the findings from cohort A and B clinical trials have been reported in numerous publications. The data from this trial indicated that a single cilta-cel infusion resulted in early and deep responses with a manageable safety profile in patients with progressive disease after 1-3 prior LOT (cohort A) and in those who experienced early relapse or treatment failure after initial therapy (cohort B) (48, 54). The patient’s responses to cilta-cel have been demonstrated to further deepen over time in the consecutive follow-up data. Besides, the approach of cilta-cel usage in outpatient settings is being explored in the CARTITUDE-2 study and the published results revealed that outpatient dosing of cilta-cel may be feasible (45, 47, 48). This is supported by the reports from CARTITUDE-1, indicating the possibility of using cilta-cel as part of outpatient treatment based on its findings of a low rate of grade 3 CRS, 7.0 days of median time to CRS onset, and 4 days of median duration (69). Cumulatively, CARTITUDE-2 indicates that cilta-cel exhibits promising antimyeloma activities in different case scenarios of MM, which encourages further research into cilta-cel along with previous LOT and its incorporation into potentially curative frontline regimens. This gives the cancer community tremendous hope that cilta-cel may soon be licensed for use in other MM groups and perhaps even in outpatient settings.

The CARTITUDE-2 trial is an ongoing study that is expected to continue until February 2026. Thus, other cohorts of the CARTITUDE-2 trial, such as cohort C, D, E, and F may come up in the future with new findings regarding cilta-cel use in patients with MM under various conditions. Cohort C of the CARTITUDE-2 study is currently in progress to assess the efficacy and safety of cilta-cel in r/r MM after PI, anti-CD38 antibody, an IMiD, and BCMA-directed treatment. However, the study results of cohort C trial have not yet been published or reported. Similarly, cohort D, E, and F are also currently active with different aims regarding cilta-cel utilization in MM (70).

In addition, cilta-cel is under study in the CARTITUDE-4 clinical trial to assess its outcomes in relapsed and lenalidomide-refractory MM. CARTITUDE-4 (NCT04181827 or MMY3002) is a phase 3, randomized, open-label study that is currently underway to compare the safety and efficacy of cilta-cel versus standard therapies like pomalidomide, bortezomib, and dexamethasone or daratumumab, pomalidomide, and dexamethasone in adult patients with relapsed and lenalidomide refractory MM. Although the clinical trial is not yet recruiting patients, it is anticipated to conduct among 400 patients from roughly 100 clinical sites in 17 countries under the sponsor of Janssen and J&J (71). Janssen Research and Development has also launched another phase 3, randomized, open-label, multicenter, global study on cilta-cel, known as CARTITUDE-5 (MMY3004 or NCT04923893). This trial is targeted to enroll 650 patients from 118 clinical centers in 25 countries across the globe, with the aim to assess the efficacy and safety of cilta-cel as first-line therapy in patients with newly diagnosed MM not intended for transplant (72). Furthermore, CARTITUDE-6 (MMY3005; NCT05257083) is another ongoing clinical trial on cilta-cel in patients with newly diagnosed MM. It is also a phase 3, randomized, open-label, international study aimed to involve 750 patients from about 52 clinical sites (multicenter) to compare the efficacy and safety of DVRd (daratumumab, bortezomib, lenalidomide, and dexamethasone) followed by a single infusion of cilta-cel (target dose of 0.75×10^6^ CAR-positive viable T-cells/kg) versus DVRd followed by autologous stem cell transplant (ASCT) in patients with newly diagnosed MM who are not exposed to prior BCMA targeted therapy (73). All in all, these ongoing clinical trials may provide more room for the improvement of cilta-cel use in MM patients under various conditions and settings where it is not approved for medical use by any of the regulatory agencies.

## Future perspectives

Jansen and Legend Biotech jointly first developed and commercialize cilta-cel, which was approved to be used as a frontier LOT for r/r MM. It is now part of the treatment arsenal of MM, inducing deep and durable responses in heavily pre-treated myeloma patients who have no other alternatives. But the long-term safety of cilta-cel is still waiting for a post-marketing observational study. Further research is also required to enhance the anti-tumor activity and lower the potential associated toxicity of this therapy which can provide new insights into the effectiveness and future advances of this therapeutic strategy. Along with ide-cel, cilta-cel is believed to drastically improve the therapeutic landscape of MM and create huge excitement among the wider oncology community. However, challenges may be created in the future among oncologists in selecting between the two therapies for patients with r/r MM. They both are demonstrated to enhance patient outcomes impressively and are associated with non-severe, manageable adverse effects. But a crude comparison between the efficacy outcomes and safety profiles from their respective clinical trials demonstrated that cilta-cel has better efficacy than ide-cel. A more recent study using the MAIC method also indicates that cilta-cel has superior efficacy than ide-cel. This suggests that cilta-cel may be the first choice than ide-cel for r/r MM patients. However, some scholars also hypothesized that ide-cel has a very good outcome and certain types of patients may be better suited for ide-cel than cilta-cel, including those with baseline neurological diseases like polyneuropathies or movement disorders. Thus, more data are required to have sufficient evidence regarding the efficacy and adverse events, as well as to decide the treatment choice between the two CAR T-cell products in treating MM patients.

Currently, numerous clinical trials are being accrued to further examine the safety and efficacy of cilta cel in the treatment of different MM conditions. Published results of some of these ongoing trials showed excellent efficacy and a low rate of adverse events, encouraging cilta-cel usage in MM under a variety of settings in the future (51). It is well known that a combination of multiple treatment approaches targeting different mechanisms is a more effective way of disease management. However, there have been only a few studies examining BCMA-directed CAR T-cell treatment in combination with a wide range of anti-myeloma agents. Lenalidomide was demonstrated to be able to increase the durability and activity of CAR T-cells in preclinical trials (74, 75). Similarly, some clinical studies are currently in progress to investigate the safety and efficacy of cilta-cel-based combination therapies in different MM circumstances. Though earlier results showed its promising antimyeloma activities, cilta-cel alone or in combination with other LOT has not yet received approval by any regulatory agencies, including FDA, for clinical use in MM other than the recently approved ones. This indicates that the ongoing clinical trials are highly awaited to lend FDA approval of cilta cel in other MM types.

In addition to MM, BCMA plays a key role in the development of other B-cell malignancies, such as leukemia and lymphomas. This may provide insights that cilta-cel or other BCMA-targeting CAR T-cell therapy may potentially use as alternative treatment approaches in various blood cancers beyond MM. Thus, investigating cilta-cel or other anti-BCMA-CAR T-cell agents for therapeutic use in other hematological malignancies other than MM in the hope of discovering new therapeutic options for patients with lymphoma and leukemia needs to be considered. Moreover, reprogramming the current design of CAR T-cell to the advanced generation of CAR can also have the utmost importance to further improve the current outlook of MM therapy. Indeed, this is currently under active development to transform the treatment paradigm of MM into a more advanced platform. Overall, although CAR T-cell therapy in MM has shown outstanding outcomes and an exciting advancement, it is still in its infancy that warrants further research to find more effective anti-myeloma agents.

## Concluding remarks

Conclusively, cilta-cel is a BCMA-directed, genetically modified autologous T-cell therapy, which involves reprogramming a patient’s own T-cells with a transgene encoding a synthetic CAR receptor that identifies and eliminates BCMA expressing cancer cells. This is a second-generation CAR T-cell product that carries the characteristics of a TCR containing a CD3ζ signaling domain and a 4-1BB costimulatory domain as well as mAbs targeting BCMA. Cilta-cel was approved for the treatment of patients with r/r MM whose disease has come back or no longer responds after initial therapy with at least four prior triple-class therapies, such as a PI, an IMiD, and an anti-CD38 mAb. It has shown unprecedented outcomes in heavily pretreated patients, yielding early, deep, and durable responses among patients with r/r MM, with a tolerable safety profile. It is nowadays incorporated into potentially curative frontline regimens as part of the diverse portfolio of anti-myeloma agents for very resistant MM. Cilta-cel is also superior to ide-cel in terms of its therapeutic efficacy in treating r/r MM patients. Several studies are currently in progress to further investigate cilta-cel safety and efficacy in the treatment of MM under various settings.

## Author contributions

All authors made a significant contribution to the work reported, whether that is in the conception, study design, execution, acquisition of data, analysis and interpretation, or in all these areas, took part in drafting, revising or critically reviewing the article, gave final approval of the version to be published, have agreed on the journal to which the article has been submitted, and agree to be accountable for all aspects of the work.

## Conflict of interest

The authors declare that the research was conducted in the absence of any commercial or financial relationships that could be construed as a potential conflict of interest.

## Publisher’s note

All claims expressed in this article are solely those of the authors and do not necessarily represent those of their affiliated organizations, or those of the publisher, the editors and the reviewers. Any product that may be evaluated in this article, or claim that may be made by its manufacturer, is not guaranteed or endorsed by the publisher.

## Abbreviations

ASCT, autologous stem cell transplant; BAFF, B-cell activating factor; BCMA, B cell maturation antigen; CAR, Chimeric Antigen Receptor; CM, costimulatory molecule; CR, complete response rate; CRS, Cytokine Release Syndrome; CRS, cytokine release syndrome; DoR, duration of response; DVRd, daratumumab, bortezomib, lenalidomide, and dexamethasone; FDA, Food and Administration; ICANS, immune effector cell-associated neurotoxicity syndrome; IL-2-Interleukin-2; IMiDs, immunomodulatory drugs; IMWG, International Myeloma Working Group; ITAM, immunoreceptor tyrosine-based activation motif; LOT, line of therapy; mAb, monoclonal antibody; MAIC, matching-adjusted indirect comparison; MRD, Minimal residual disease; ORR, overall response rate; OS, overall survival; PFS, progression-free survival; PI, proteasome inhibitors; r/r MM, relapsed or refractory multiple myeloma; scFv, single-chain variable fragment; sCR, stringent complete response rate; TCR, T-Cell Receptor; TEAEs, treatment emergent adverse events; TMD, transmembrane domain; TNFRSF 17, tumor necrosis factor superfamily member 17 receptor; TRAF, tumor necrosis factor receptor-associated factor; VGPR, very good partial response.
